# Studies on the Antidiabetic and Antinephritic Activities of *Paecilomyces hepiali* Water Extract in Diet-Streptozotocin-Induced Diabetic Sprague Dawley Rats

**DOI:** 10.1155/2016/4368380

**Published:** 2016-02-29

**Authors:** Juan Wang, Lirong Teng, Yange Liu, Wenji Hu, Wenqi Chen, Xi Hu, Yingwu Wang, Di Wang

**Affiliations:** School of Life Sciences, Jilin University, Changchun 130012, China

## Abstract

*Paecilomyces hepiali* is a fungus widely used in Asian countries for various potential pharmacological activities. The present study aims to evaluate the antidiabetic and antinephritic effects of the* Paecilomyces hepiali* mycelium water extract (PHC) in diabetic rat, which is established by eight-week high-fat diet administration followed by one-week tail intravenous injection of 25 mg/kg streptozotocin (STZ). After four-week 0.12 g/kg metformin and PHC at doses of 0.08, 0.4, and 2.0 g/kg treatment, an increment of body weight, a decrement of plasma glucose, low levels of total cholesterol, and low density lipoprotein cholesterol in diabetic rats were observed. PHC promotes glucose metabolism by enhancing insulin, pyruvate kinase activity, and increasing the synthesis of glycogen. PHC normalized the disturbed levels of superoxide dismutase, methane dicarboxylic aldehyde, and glutathione peroxidase in kidney. The inhibitory effects on the levels of interleukin-2, interleukin-6, interleukin-10, and tumor necrosis factor-*α* in serum and kidney revealed the protection of PHC against diabetic nephropathy. Compared with nontreated diabetic rats, four-week PHC treatment resulted in a decrement on nuclear factor kappa B expression in kidney. These results show that* Paecilomyces hepiali* possesses antidiabetic and antinephritic effects which are related to the modulation of nuclear factor kappa B activity.

## 1. Introduction 

Diabetes, characterized by hyperglycemia and metabolic disturbance on lipids, carbohydrates, and proteins, affect the life quality of patients by bringing huge pressure to society and public health [1]. Nearly 2.2% of total death in the world is caused by diabetes [2]. Type II diabetes, considered as the common form of diabetes, will affect the health of 8 billion people in the world till 2025 [3]. Persistent hyperglycemia in diabetes mellitus leads to the development of secondary complications including neuropathy, nephropathy, and retinopathy [2].

Diabetic nephropathy is the major cause of end-stage renal disease with high mortality and morbidity [4]. A major clinical manifestation of diabetic nephropathy is that microalbuminuria follows macroalbuminuria and further leads to renal dysfunction [5]. During this process, a number of key pathways, including advanced glycation and activation of intracellular signalling molecules, are involved [6]. According to statistics, diabetic nephropathy accounted for over 25% of the incident patients with end-stage renal disease (ESRD) in 2013 in the United Kingdom; meanwhile, in the United States, over 40% diabetic nephropathic patients received dialysis [7].

Recently, no satisfactory therapeutic regimens can cure diabetes although most of them have normalized blood glucose and fat levels and improved microcirculation [8]. Traditional treatment only focuses on pancreatic islet function recovery and blood glucose regulation. Additionally, some oral antihyperglycemic agents display various adverse effects including hypoglycemia, edema, gastrointestinal disturbances, and insulin resistance [9]. Searching for alternative treatment of diabetes and related complications is highly demanded.

Herbs turn out to be a valuable reservoir for novel drugs due to the potent efficacy with few side effects [10].* Cordyceps sinensis*, one of the most well-known traditional Chinese medicines and folk tonic food, is commonly used for prevention and treatment of a variety of diseases, such as anti-oxidation, anti-tumor, immunomodulatory, and hypoglycemic activities [11]. It has been demonstrated that* Paecilomyces hepiali,* a derivative from* Cordyceps sinensis*, shows anticancer and anti-type I diabetic properties [12, 13]. As reported, polysaccharides-enriching* Paecilomyces hepiali* water extract induces A549 cell apoptosis via TNF-*α* related pathway [12] and displays rental protective activity in adriamycin-induced nephropathy rat models [14]. In our group, it has been verified in the separated experiments that* Paecilomyces hepiali* water extracts displayed antifatigue, antihypoxia, and antidepressant-like effects in relevant mouse or rat models. The potential regulatory effect of* Paecilomyces hepiali* on diet-streptozotocin- (STZ-) induced type II diabetic rat has not been reported yet.

We therefore hypothesized that polysaccharides-enriching* Paecilomyces hepiali* water extract (PHC) may possess antidiabetic property. In the present study, a high-fat diet and STZ-induced rat model was applied to observe the effects of PHC on diabetes and renal injury and its possible mechanisms involving nuclear factors kappa B (NF-*κ*B) associated with inflammatory activation events.

## 2. Materials and Methods

### 2.1. *Paecilomyces hepiali* Water Extract Preparation

*Paecilomyces hepiali* mycelium obtained from submerged fermentation was extracted at 80°C for 4 h in double distilled (DD) water twice [15]. After the centrifugation at 5,000 rpm for 10 min, the supernatant was sequentially concentrated in an evaporator under reduced pressure and freeze-dried conditions to produce the solid aqueous extract (PHC). Preliminary determination showed that PHC contains 9.8% polysaccharides, 15.5% total proteins, 5.9% organic acid, and 0.4% adenosine.

### 2.2. Animal Care

The experimental animal protocol was approved by the Animal Ethics Committee of Jilin University. Male Sprague Dawley rats (8 weeks; 180 g–220 g) (SCXK(JI)-2011-0003) were housed under standard laboratory conditions of 23°C ± 1°C, relative humidity of 55%, and 12-h : 12-h light/dark cycle (lights on 7:00–19:00 h) during the study. The animals were given standard rat pellets and tap water* ad libitum*. All efforts were made to minimize animal suffering and to reduce the number of animals used.

### 2.3. Diet-Streptozotocin-Induced Diabetic Rat Model and Drug Administration Procedure

The rats were randomly divided into two groups and fed with either the standard control diet (normal control group, *n* = 10) or a high-fat diet (HFHSD, 12% protein, 5% fat, 67% carbohydrate, 5% cholesterol, and 5% other additives) (*n* = 50) for 8 weeks [16]. HFHSD-treated rats were further injected with 25 mg/kg streptozotocin (STZ) agent dissolved in a citrate buffer (0.1 mol/L sodium citrate and 0.1 mol/L citric acid, pH 4.5) for one week. Rats with a blood glucose level of more than 11.1 mmol/L were defined as having diabetes after the last STZ injection for 72 h.

Successfully established type II diabetic rats were divided into five groups randomly, and they were orally treated with 2.0 mL/kg sterile saline (model group, *n* = 10), 0.12 g/kg metformin hydrochloride (Met; purchased from Beijing Jingfeng Zhiyao Co., Ltd., Beijing, China) (positive control group, *n* = 10), 0.08 g/kg PHC (low drug-treated group, *n* = 10), 0.4 g/kg PHC (middle drug-treated group, *n* = 10), and 2.0 g/kg PHC (high drug-treated group, *n* = 10). Normal rats, which were given 2.0 mL/kg sterile saline, served as normal control group. Drug delivery time lasted for four weeks. During the whole experiment, bodyweight and blood glucose were recorded every week.

### 2.4. Oral Glucose Tolerance Test (OGTT) in Rats

After the last drug administration, all the rats were fasted for 16 h and a glucose tolerance test was performed. Briefly, rats were weighted and then orally given glucose (2.0 g/kg). Tail-vein blood samples were collected at different time from 0 to 240 min and then were assayed by using the fast blood glucose meter [17]. Calculation of the area under the blood glucose curve (AUC) was made according to I [15]

IAUC=basal glycaemia+glycaemia 0.5 h×0.25+glycaemia 0.5 h+glycaemia 1 h×0.25+glycaemia 1 h+glycaemia 2 h×0.5.

### 2.5. Samples Collection and Biochemical Indexes Analysis

Before sacrifice, blood was sampled from the heart of all the rats under anesthesia. Heart, spleen, kidney, and liver tissues were quickly collected, weighted, and stored in liquid nitrogen. The levels of insulin (INS) in serum, interleukin-2 (IL-2), interleukin-6 (IL-6), interleukin-10 (IL-10), and tumor necrosis factor-*α* (TNF-*α*) in serum and kidney were detected with related enzyme-linked immunosorbent assay (ELISA) kits (Calbiotech, USA). The concentrations of glycosylated hemoglobin (HbA1c), pyruvate kinase (PK), low density lipoprotein cholesterol (LDL-C), total cholesterol (T-CHO), albumin in serum, superoxide dismutase (SOD), methane dicarboxylic aldehyde (MDA), glutathione peroxidase (GSH-Px) in serum and kidney, and glycogen in liver were determined by using commercial kits obtained from Nanjing Biotechnology Co., Ltd. (Nanjing, China).

### 2.6. Western Blot

One part of collected kidney was homogenized in radioimmunoprecipitation assay buffer (RIPA, Sigma-Aldrich, USA) containing 1% protease inhibitor cocktail (Sigma-Aldrich, USA) and 2% phenylmethanesulfonyl fluoride (PMSF, Sigma-Aldrich, USA). Protein concentrations were determined by Bradford method, and 40 *μ*g proteins were separated by using a 12% SDS-PAGE gel and transferred electrophoretically onto nitrocellulose membranes (0.45 *μ*m; Bio Basic, Inc. USA). The transferred membranes were blotted with primary antibodies at 4°C overnight at dilution of 1 : 500, nuclear factor-*κ*B (NF-*κ*B) receptor and glyceraldehyde-3-phosphate dehydrogenase (GAPDH) (Santa Cruz, USA), and then they were incubated with horseradish peroxidase-conjugated secondary antibodies (Santa Cruz, USA). Chemiluminescence was detected by using ECL detection kits (GE Healthcare, UK). The intensity of the bands was quantified by scanning densitometry using software Image J.

### 2.7. Statistical Analysis

All values were expressed as mean ± SD. One-way analysis of variance (ANOVA) was used to detect statistical significance followed by* post hoc* multiple comparisons (Dunn's test). A value of *P* < 0.05 was considered to be significant.

## 3. Results 

### 3.1. The Hypoglycemic Effect of PHC on Diet-STZ-Induced Diabetic Rats

**Figure 1:**
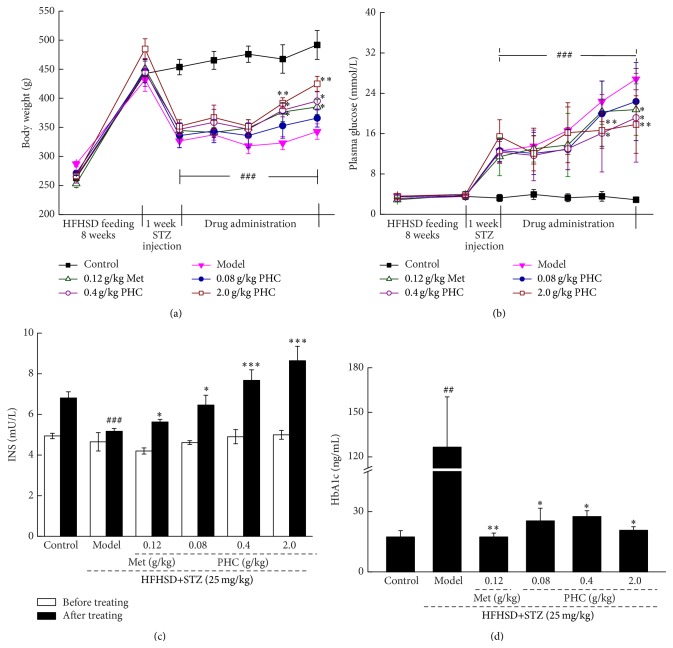
Diet-STZ-induced diabetic rats were treated with or without 0.12 g/kg metformin (Met) and* Paecilomyces hepiali* water extract (PHC) at indicated doses for four weeks. The changes on body weight (a) and fasting plasma glucose level (b) were monitored during the whole drug administration period. After the final drug treatment, the serum levels of insulin (c) and glycosylated hemoglobin (d) were detected in all experimental rats. Data are expressed as mean ± SD (*n* = 10) and analyzed by using one-way ANOVA. ^##^ *P* < 0.01 and ^###^ *P* < 0.001 versus normal controls, ^*∗*^ *P* < 0.05, ^*∗∗*^ *P* < 0.01, and ^*∗∗∗*^ *P* < 0.001 versus nontreated diabetic rats.

The strikingly reduced bodyweight and enhanced blood glucose were observed after STZ injection in diet-STZ-induced diabetic rats (*P* < 0.001, Figures 1(a) and 1(b)). Four-week treatment of 120 mg/kg Met strongly reversed these abnormal changes (*P* < 0.05, Figures 1(a) and 1(b)). Similar to Met, compared with model group, the maximum increment of bodyweight was nearly 23.9% in PHC-treated diabetic rats (*P* < 0.01, Figure 1(a)). PHC administration at dose of 0.4 and 2.0 g/kg resulted in 27.1% and 34.9% reduction on fasting blood glucose compared with nontreated diabetic rats (*P* < 0.05, Figure 1(b)). Additionally, 28-day PHC treatment significantly increased the hypolevel of serum insulin which was caused by high-fat diet feeding and STZ injection (*P* < 0.05, Figure 1(c)). The extremely high level of HbA1c in diabetic rats was suppressed by PHC, and an 83.7% reduction was found in 2.0 g/kg of PHC-treated rats (*P* < 0.05, Figure 1(d)).

**Figure 2:**
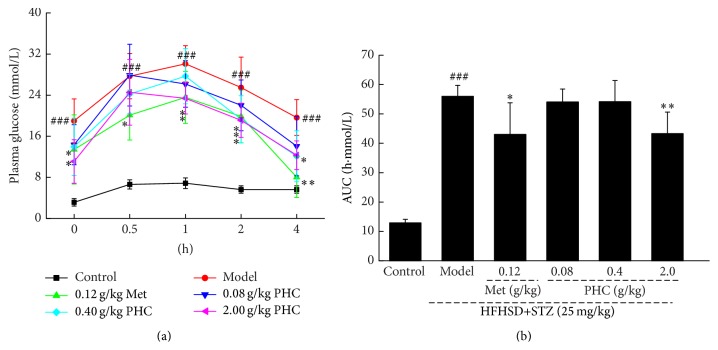
An oral glucose tolerance test was further performed to avoid a false positive result. After an oral administration of 2 g/kg D-glucose in all experimental rats, the changes of plasma glucose (a) and area under the curve of glucose (b) were analyzed. Data are expressed as mean ± SD (*n* = 10) and analyzed by using one-way ANOVA. ^###^ *P* < 0.001 versus control, ^*∗*^ *P* < 0.05 and ^*∗∗*^ *P* < 0.01 versus model group.

OGTT was applied to avoid false positive results obtained from the levels of blood glucose and HbA1c. The level of fasting blood glucose was extremely higher in model rats than that in normal control rats (*P* < 0.001, Figure 2(a)). Within 30 min of OGTT starting, blood glucose concentration was almost doubled compared with its initial control value. 2.0 g/kg PHC treatment significantly prevented blood glucose levels from shooting up, especially at the time point of 60, 120, and 240 min (*P* < 0.05, Figure 2(a)). The suppressive effects of PHC on fasting blood glucose level were further confirmed by AUC calculation.

### 3.2. The Regulatory Effects of PHC on PK and Glycogen in Diabetic Rats

**Figure 3:**
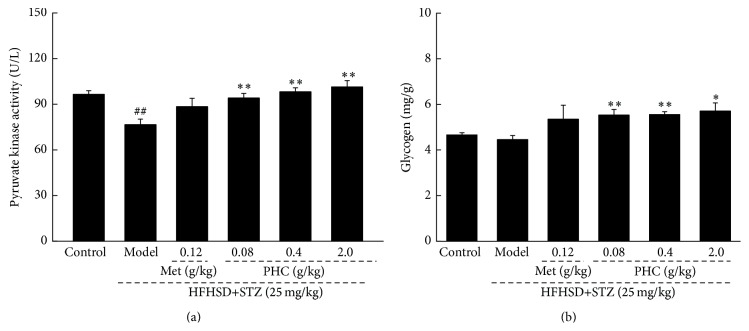
After four-week PHC oral administration, the levels of pyruvate kinase in serum (a) and glycogen in liver (b) were significantly enhanced compared with nontreated diabetic rats. Data are expressed as mean ± SD (*n* = 10) and analyzed by using one-way ANOVA. ^##^ *P* < 0.01 versus control, ^*∗*^ *P* < 0.05 and ^*∗∗*^ *P* < 0.01 versus model group.

PK, recognized as a rate-limiting enzyme of glycolytic pathway, promotes the metabolism of sugar [18]. Compared with nontreated diabetic rats, up to 32.5% increment of serum PK concentration was noted in 2.0 g/kg PHC-treated diabetic rats (*P* < 0.01, Figure 3(a)). Additionally, 0.08, 0.4, and 2.0 g/kg PHC administration resulted in 24.1%, 24.6%, and 28.1% enhancement of hepatic glycogen level in diabetic model rats, respectively (*P* < 0.05, Figure 3(b)).

### 3.3. The Hypolipidemic Effect of PHC in Diabetic Rats

**Figure 4:**
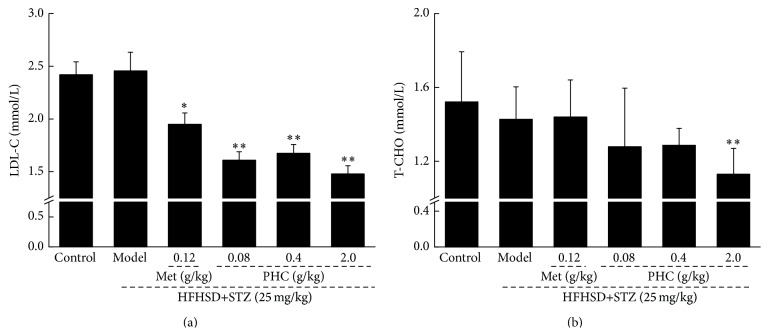
Four-week Met and PHC treatment strongly reduced the serum levels of LDL-C (a) and T-CHO (b) in diet-STZ-induced diabetic rats. Data are expressed as mean ± SD (*n* = 10) and analyzed by using one-way ANOVA. ^*∗*^ *P* < 0.05 and ^*∗∗*^ *P* < 0.01 versus model group.

After four-week PHC treatment, the significant reduction of serum levels of LDL-C and T-CHO was observed in experimental rats (*P* < 0.01, Figure 4). Different from PHC, Met treatment only normalized the serum concentration of LDL-C rather than T-CHO (Figure 4).

### 3.4. Antinephropathic Effect of PHC in Diabetic Rats

The enhanced organ indexes of liver, kidney and the decrement of spleen were noted in diabetic model rats (*P* < 0.05; Table 1). Only PHC treatment normalized the indexes of liver and kidney to a healthy level (*P* < 0.05; Table 1).

A significant increment of serum and kidney levels of IL-2, IL-6, IL-10, and TNF-*α* was observed in diet-STZ-induced diabetic rats, which was relieved by four-week PHC treatment (*P* < 0.05; Table 2). Interestingly, Met administration only influenced the serum concentration of IL-6 and IL-10 and IL-2 in kidney of diabetic rats (*P* < 0.05; Table 2).

**Figure 5:**
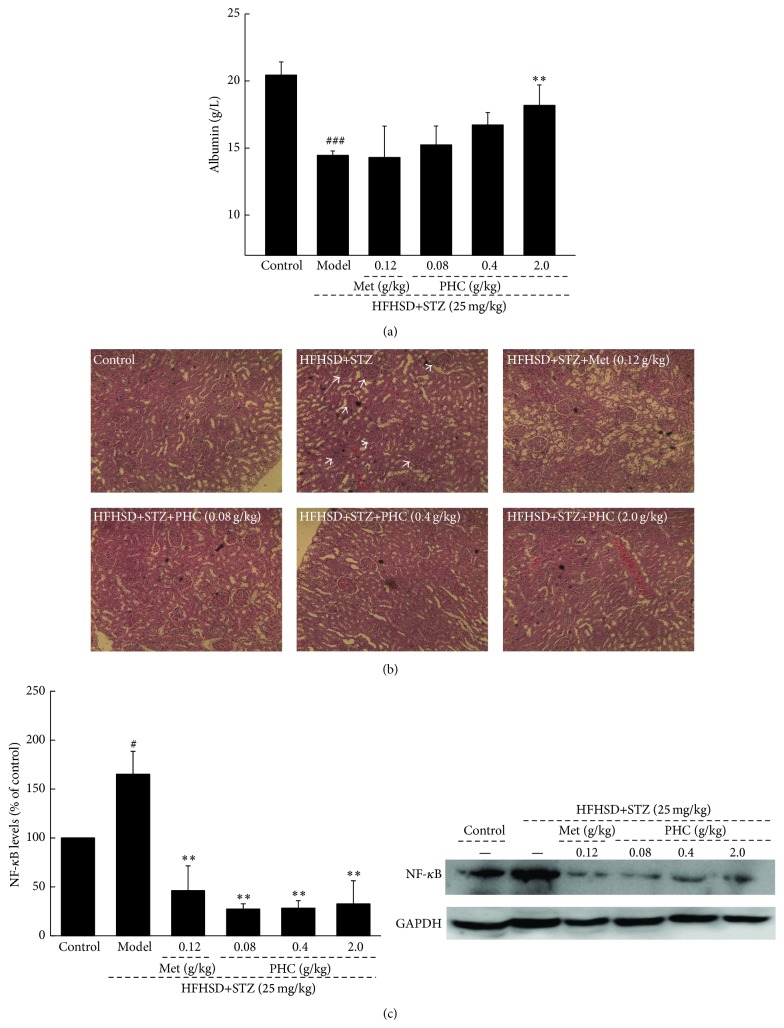
Diet-STZ-induced diabetic rats were orally treated with or without Met and PHC for four weeks. (a) PHC enhanced the low serum level of albumin. (b) Histopathological analyses in kidney were applied via H&E staining (×100). (c) The expression of NF-*κ*B in kidney was analyzed via western blot. Quantification data of the expressions of NF-*κ*B was normalized by corresponding GAPDH. Data are expressed as mean ± SD (*n* = 10) and analyzed by using one-way ANOVA. ^#^ *P* < 0.05 and ^###^ *P* < 0.001 versus control, ^*∗∗*^ *P* < 0.01 versus model group.

Overexpression of NF-*κ*B and a strong reduction of albumin were observed in diet-STZ-induced diabetic rats, which were all relieved by four-week PHC treatment (*P* < 0.05; Figure 5). Compared with nontreated diabetic rats, up to 25.7% increment of serum albumin concentration was noted in 2.0 g/kg PHC-treated diabetic rats (*P* < 0.01, Figure 5(a)). Additionally, 0.08, 0.4, and 2.0 g/kg PHC administration resulted in 83.5%, 82.9%, and 80.3% decrement of NF-*κ*B expression in diabetic model rats, respectively (*P* < 0.01, Figure 5(c)). The incidences of glomerular basement membrane thickening, mesangial proliferation, and inflammatory infiltrate injuries were noted in kidney tissue of diet-STZ-induced diabetic rats, and they were significantly ameliorated by Met and PHC (Figure 5(b)).

### 3.5. The Antioxidant Parameters of PHC on Diabetic Rats

GSH-Px and SOD play important roles in preventing oxidative injury on animals [19, 20]. Overproduction of MDA and hypoactivities of SOD and GSH-Px were observed in serum and kidney of diet-STZ-induced diabetic rats compared with normal control group (*P* < 0.05; Table 3). Different from the antioxidative effect of Met, PHC at dose of 2.0 g/kg resulted in a 33.5% reduction on MDA level, and 85.4% and 113.2% increment on SOD and GSH-Px activities compared with nontreated diabetic rats (*P* < 0.05; Table 3). However, no significant influence on serum levels of SOD, GSH-Px, and MDA was noted in PHC-treated diabetic rats.

## 4. Discussion

Via inducing selective pancreatic islet *β*-cell cytotoxicity, STZ is commonly used to develop experimental diabetic animal models [21]. Type II diabetes mellitus is characterized by insufficient insulin secretion and insulin resistance [22]. Our present study aims to investigate the effect of PHC on diabetes mellitus and related mechanism in high-fat diet/STZ-induced type II diabetic rat models. Similar to our results, STZ treatment also caused a low level of bodyweight and hyperconcentration of HbA1c [21]. Compared with nontreated diabetic rats, PHC administration normalized the bodyweight and serum HbA1c level and further enhanced the low insulin secretion. Combining with the suppressive activity of PHC on hyperlevel of fasting blood glucose in diabetic rats, the hypoglycemic property of PHC was observed. PHC-mediated antidiabetic activity is verified by oral glucose tolerance test (OGTT), which is a more sensitive measure of early abnormalities in glucose regulation than fasting plasma glucose or HbA1c [2].

Abnormal changes on glucose metabolism, including decreased glycolysis, impeded glycogenesis, and increased gluconeogenesis in diabetic patients, are observed [23]. Pyruvate kinase couples the free energy of phosphoenolpyruvate hydrolysis for ATP synthesis to form pyruvate [24]. Glycogen is the primary intracellular storable form of glucose, and its level in liver and skeletal muscles directly reflects the activity of insulin [25]. As an important storage material, glycogen is a source of readily available glucose for living organisms [26]. The synthesis and degradation of glycogen, which are controlled by glucose level, are considered as reciprocally regulated pathways [27]. On STZ-induced diabetic rats, the selective destruct *β*-cells of pancreas islet lead to the reduction of insulin level, which further results in the suppression of glycogen in tissues [28]. The enhanced PK concentration in serum and glycogen level in the liver of PHC-treated diabetic rats further confirmed the antidiabetic activity of PHC.

It is a great risk for diabetes patients to develop atherosclerosis and coronary artery disease [29]. Lipids are indicated as one of the major pathogenic biological markers in situations of metabolic dysfunction [30]. Four-week PHC administration strongly reduced serum levels of LDL-C and T-CHO in established diabetic rats which indicates* Paecilomyces hepiali* possesses hypolipidemic effect.

Diabetic nephropathy is one of the most common microvascular complications of diabetes mellitus [31], which begins with glomerular hyperfiltration caused by hyperglycemia, and further results in glomerular hypertrophy and glomerular basement membrane thickening [32]. Anti-inflammatory and immunosuppressive factors play important roles during diabetic nephropathy treatment. IL-10 is recognized as an anti-inflammatory cytokine limiting the cascade of proinflammatory cytokines [33]. IL-2 can indirectly induce the expression of endogenous cytokines, such as INF-g [34]. It has been confirmed that the neural damage is related with the elevation of proinflammatory cytokines which includes IL-6 and TNF-*α* [35]. Previous study demonstrates that IL-6 influences B cell development [36]. PHC not only normalized the serum and kidney levels of IL-2, IL-6, IL-10, and TNF-*α*, but also regulated serum albumin concentration in diabetic rats [37]. Under proteinuric conditions, albumin serves as one of pathogeneses in chronic tubulointerstitial damage [38]. Albumin is essential for maintaining the oncotic pressure which is needed for proper distribution of body fluids between blood vessels and body tissues. Glycated albumin regulates VEGF expression to promote proteinuria and glomerulosclerosis in diabetes, suppresses insulin secretion in pancreatic *β*-cells, and stimulates cultured retinal microglia to secrete inflammatory cytokines [39]. Additionally, the activity of NF-*κ*B played an important role for renal protection via promoting transcription of proinflammatory cytokine [40–42]. As reported, Simiao pill activated Sirt1 to suppress inflammatory responses through inhibition of NF-*κ*B/NLRP3 inflammasome activation and further to improve glomerular function to resolute proteinuria [17]. Our data showed that through NF-*κ*B inhibition PHC is able to suppress the release of proinflammatory cytokines, including TNF-*α*, IL-2, IL-6, and IL10.

Furthermore, chronically high blood glucose level disrupts the antioxidant system of tissues [43]. Enzymatic antioxidants, such as SOD and GSH-Px, have been considered as primary enzymes since they were involved in the direct elimination of reactive oxygen species (ROS) [44]. MDA is thought to be an indicator of the lipid peroxidation process [45]. Four-week PHC administration has successfully normalized the disordered levels of SOD, GSH-Px, and MDA in kidney of diabetic rats. However, further investigation is still necessary for the detailed roles of oxidative system on PHC-mediated antidiabetic and antinephritic activities.

In conclusion, in high-fat diet/STZ-induced type II diabetic rat models, we successfully confirmed the antidiabetic and antinephritic properties of PHC indicated by decreasing fasting plasma glucose level, enhancing the glycometabolism, and balancing the state of oxidative system. Its hypolipidemic activity was also observed in the present study. Further data revealed that the modulation on the activation of NF-*κ*B may play the central role upon these effects. Although further more experiments are needed to investigate the in-depth pharmacological mechanisms, our results provide an evidence for* Paecilomyces hepiali* to be used as an antidiabetic and antinephritic agent.

## Figures and Tables

**Table 1:** The effects of Met and PHC on the ratio of organ and body weight in diabetic rats were analyzed.

	Control	Model	0.12 g/kg Met	0.08 g/kg PHC	0.4 g/kg PHC	2.0 g/kg PHC
Heart (%)	0.35 ± 0.05	0.31 ± 0.05	0.34 ± 0.05	0.31 ± 0.03	0.32 ± 0.05	0.30 ± 0.02
Liver (%)	2.59 ± 0.20	3.74 ± 0.38^###^	3.80 ± 0.28	3.50 ± 0.48	3.46 ± 0.24	3.22 ± 0.27^*∗∗*^
Spleen (%)	0.42 ± 0.14	0.32 ± 0.07^#^	0.38 ± 0.05	0.33 ± 0.06	0.35 ± 0.11	0.32 ± 0.11
Kidney (%)	0.64 ± 0.08	1.08 ± 0.14^###^	1.18 ± 0.19	1.01 ± 0.08	1.04 ± 0.08	0.97 ± 0.05^*∗*^

Data are expressed as mean ± SD (*n* = 10) and analyzed by using one-way ANOVA. ^#^ *P* < 0.05 and ^###^ *P* < 0.001 versus normal controls, ^*∗*^ *P* < 0.05 and ^*∗∗*^ *P* < 0.01 versus model group.

**Table 2:** The regulatory effects of Met and PHC on the inflammatory related factors in diabetic rats.

		Control	Model	0.12 g/kg Met	0.08 g/kg PHC	0.4 g/kg PHC	2.0 g/kg PHC
Serum	IL-2 (pg/mL)	67.77 ± 3.08	89.10 ± 6.06^#^	83.93 ± 14.03	76.1 ± 3.27	72.77 ± 5.91	72.1 ± 1.71^*∗*^
	IL-6 (pg/mL)	6.51 ± 0.24	7.28 ± 0.23^#^	5.04 ± 0.31^*∗∗*^	5.59 ± 0.36^*∗∗*^	5.54 ± 0.32^*∗∗*^	5.29 ± 0.32^*∗∗*^
	IL-10 (pg/mL)	3.32 ± 0.19	5.20 ± 0.47^##^	3.75 ± 0.27^*∗*^	3.94 ± 0.16^*∗*^	4.39 ± 0.21	3.56 ± 0.14^*∗∗*^
	TNF-*α* (pg/mL)	9.88 ± 0.55	12.15 ± 0.37^##^	11.12 ± 0.72	11.15 ± 1.04	9.75 ± 0.65^*∗∗*^	8.96 ± 0.68^*∗∗*^

Kidney	IL-2 (pg/g)	3394 ± 735	6932 ± 344^##^	4065 ± 1060^*∗*^	2804 ± 437^*∗∗*^	3189 ± 265^*∗∗*^	3101 ± 754^*∗∗*^
	IL-6 (pg/g)	10.86 ± 1.29	72.73 ± 17.88^##^	30.17 ± 7.31	46.18 ± 5.44	47.49 ± 7.35	17.53 ± 2.78^*∗*^
	IL-10 (pg/g)	27.78 ± 2.66	40.66 ± 5.33^#^	28.41 ± 3.60	27.65 ± 2.05^*∗*^	28.91 ± 3.14	23.90 ± 2.25^*∗∗*^
	TNF-*α* (pg/g)	283.96 ± 38.16	416.3 ± 28.7^#^	328.30 ± 76.23	223.21 ± 29.68^*∗∗*^	225.66 ± 26.57^*∗∗*^	158.49 ± 41.27^*∗∗*^

The levels of IL-2, IL-6, IL-10, and TNF-*α* in serum and kidney were determined and detected after 4-week administration of Met (0.12 g/kg) or PHC (0.08, 0.4, and 2.0 g/kg). Data are expressed as mean ± SD (*n* = 10) and analyzed by one-way ANOVA. ^#^ *P* < 0.05 and ^##^ *P* < 0.01 versus normal controls, ^*∗*^ *P* < 0.05 and ^*∗∗*^ *P* < 0.01 versus model group.

**Table 3:** The regulatory effects of Met and PHC on the oxidative damage related factors in diabetic rats.

		Control	Model	0.12 g/kg Met	0.08 g/kg PHC	0.4 g/kg PHC	2.0 g/kg PHC
Serum	MDA (nmol/mL)	11.78 ± 2.49	17.63 ± 2.21	5.87 ± 1.19^*∗∗*^	15.11 ± 1.17	14.56 ± 3.71	12.73 ± 2.45
	SOD (U/mL)	55.72 ± 2.49	50.77 ± 1.89	60.04 ± 1.64^*∗∗*^	55.63 ± 1.58	55.48 ± 1.92	55.94 ± 1.46
	GSH-Px (*μ*mol/L)	243.28 ± 30.62	119.40 ± 11.77^##^	218.66 ± 34.98^*∗*^	111.94 ± 11.33	134.33 ± 16.43	148.51 ± 33.30

Kidney	MDA (nmol/mgprot)	5.05 ± 0.43	7.08 ± 0.67^#^	3.94 ± 0.30^*∗∗*^	6.88 ± 0.27	6.77 ± 0.51	4.71 ± 0.30^*∗∗*^
	SOD (U/mgprot)	121.94 ± 9.03	60.15 ± 13.79^##^	75.97 ± 15.06	112.19 ± 15.06^*∗*^	99.21 ± 12.82	111.51 ± 10.66^*∗*^
	GSH-Px (*µ*mol/gprot)	6328.2 ± 518.0	3439.5 ± 776.5^#^	5910.2 ± 888.8	6806.6 ± 970.3	7348.9 ± 2108.2	7334.7 ± 958.6^*∗*^

After four-week Met and PHC treatment at indicated doses, the levels of MDA, SOD, and GSH-Px in serum and kidney were determined. Data are expressed as mean ± SD (*n* = 10) and analyzed by using one-way ANOVA. ^#^ *P* < 0.05 and ^##^ *P* < 0.01 versus normal controls, ^*∗*^ *P* < 0.05 and ^*∗∗*^ *P* < 0.01 versus model group.
